# The isolation of primary hepatocytes from human tissue: optimising the use of small non-encapsulated liver resection surplus

**DOI:** 10.1007/s10561-017-9641-6

**Published:** 2017-07-17

**Authors:** Charlotte J. Green, Catriona A. Charlton, Lai-Mun Wang, Michael Silva, Karl J. Morten, Leanne Hodson

**Affiliations:** 1 Oxford Centre for Diabetes, Endocrinology and Metabolism (OCDEM), University of Oxford, Churchill Hospital, Headington, Oxford, OX3 7LE UK; 2 Department of Cellular Pathology, Oxford University Hospitals, Oxford, UK; 3 Department of Hepatobiliary and Pancreatic Surgery, Oxford University Hospital NHS Trust, Churchill Hospital, Oxford, OX3 7LE UK; 4 Nuffield Department of Obstetrics and Gynaecology, The Women’s Centre, University of Oxford, John Radcliffe Hospital, Headley Way, Headington, Oxford, UK

**Keywords:** Hepatocyte, Liver, Human, Isolation

## Abstract

Two-step perfusion is considered the gold standard method for isolating hepatocytes from human liver tissue. As perfusion may require a large tissue specimen, which is encapsulated and has accessible vessels for cannulation, only a limited number of tissue samples may be suitable. Therefore, the aim of this work was to develop an alternative method to isolate hepatocytes from non-encapsulated and small samples of human liver tissue. Healthy tissue from 44 human liver resections were graded for steatosis and tissue weights between 7.8 and 600 g were used for hepatocyte isolations. Tissue was diced and underwent a two-step digestion (EDTA and collagenase). Red cell lysis buffer was used to prevent red blood cell contamination and toxicity. Isolated hepatocyte viability was determined by trypan blue exclusion. Western blot and biochemical analyses were undertaken to ascertain cellular phenotype and function. Liver tissue that weighed ≥50 g yielded significantly higher (*P* < 0.01) cell viability than tissue <50 g. Viable cells secreted urea and displayed the phenotypic hepatocyte markers albumin and cytochrome P450. Presence of steatosis in liver tissue or intra-hepatocellular triglyceride content had no effect on cell viability. This methodology allows for the isolation of viable primary human hepatocytes from small amounts of “healthy” resected liver tissue which are not suitable for perfusion. This work provides the opportunity to increase the utilisation of resection surplus tissue, and may ultimately lead to an increased number of in vitro cellular studies being undertaken using the gold-standard model of human primary hepatocytes.

## Introduction

A variety of cellular models have been used to investigate and understand human liver function and metabolism in health and disease. These models are important for delineating mechanisms of disease initiation and progression. Primary human hepatocytes are considered the gold standard human liver cell model; although availability of human liver tissue and difficulties in isolating viable cells potentially limits their use. Primary hepatocytes have been isolated from rodent and human liver tissue with the two-step perfusion method. In the late 1960’s Berry and Friend (1969) first described the isolation of hepatocytes from rat livers, which was adapted a few years later (Seglen 1972). The first reported isolation of human primary hepatocytes was published just over two decades ago (Guguen-Guillouzo et al. 1982). In recent years, there have been an increasing number of reports of human hepatocyte isolation using the two-step perfusion method and these studies have predominantly used fragments of resected liver tissue (usually removed due to primary or secondary tumours) (Bhogal et al. 2011; Lee et al. 2013, 2014; Pfeiffer et al. 2015; Kegel et al. 2016; Kluge et al. 2016).

From limited reported data it would appear that the ideal liver tissue specimen for two-step perfusion is a large wedge (weighing between 80 and 120 g) that is encapsulated with clearly visible blood vessels that penetrate the majority of the liver sample (Lecluyse and Alexandre 2010; Bhogal et al. 2011). However Alexandre et al. (2002) reported that when liver tissue weights were greater than 101 g then the proportion of undigested tissue left after the isolation process was significantly increased. More recently it has been suggested smaller amounts of tissue can be used (Werner et al. 2015). Many factors may influence the viability and yield of hepatocytes from human liver tissue including donor age and gender, presence of liver steatosis or fibrosis, and donor serum enzymes (Bhogal et al. 2011; Lee et al. 2014). However, Kluge et al. (2016) reported the perfusion method gave high yields of hepatocytes (9.56 × 10^6^/g tissue) with high viability (74%) in liver tissue from individuals with or without portal vein embolization. In contrast, Bhogal et al. (2011) suggested that median viability of hepatocytes isolated from healthy and disease tissue was 40%, with a total hepatocyte yield of 350,000 cells.

Prior to the establishment of the two-step perfusion method, rodent hepatocytes were isolated by a variety of methods which typically employed mechanical force to separate and isolate cells (Longmuir and Rees 1956; Jacob and Bhargava 1962). However, these methods often resulted in poor cell viability and damage to cell membranes (Berry and Simpson 1962). As an alternative, slicing of rat liver tissue followed by collagenase digest was undertaken, giving intact and viable cells (Howard et al. 1967; Howard and Pesch 1968) although reported yields were low (10% of total tissue) (Howard and Pesch 1968).

As the availability of human liver specimens that may be suitable for two-step perfusion is potentially limited we aimed to develop methodology that could be applied to all liver specimens including those not considered suitable for perfusion. In order to develop a method for hepatocyte isolation that was most likely to warrant high yields of viable cells we combined techniques and reagents from published two-step (Lecluyse and Alexandre 2010; Bhogal et al. 2011) and mechanical isolation (Longmuir and Rees 1956; Jacob and Bhargava 1962; Howard et al. 1967; Howard and Pesch 1968) methods as well as knowledge from the digestion of cells from other human tissues such as skeletal muscle.

## Methods

### Materials

All reagents were obtained from Life Technologies (Paisley, Scotland) unless otherwise stated. Collagenase IV, calcium chloride and Hanks balanced salt solution (HBSS) were purchased from Sigma-Aldrich (Dorset, UK). Type 1 collagen coated plates were from VWR (Leistershire, UK). Cell strainers were from Greiner Bio One (Stonehouse, UK). Western blot antibodies against cytokeratin 18 (CK18 (ab7797), alpha fetoprotein (AFP (ab3980), β-actin (ab6276) were from Abcam (Cambridge, UK), anti-albumin (human) (12001-05011) was from Universal Biologicals (Cambridge, UK) and the secondary antibodies goat anti-mouse (P0447 lot 00087763) and goat anti-rabbit (P0448 lot 00094764) were from Dako (California, USA). Primary antibodies we usually use at 1:1000 and secondary antibodies at 1:5000.

The bicinchoninic acid (BCA) protein assay was from Thermo Fischer Scientific (Northumberland, UK). Peroxidase-conjugated IgG antibodies were from Dako (Cambridge, UK). Urea and TG assays were from Instrumentation laboratory UK Ltd (Cheshire, UK). Type 1 collagen coated (Biocoat™) plates were from Corning (Germany).

### Ethics

Liver tissue was obtained from patients undergoing surgery who had consented (NRES Committee South Central; Berkshire B 11/SC/0443) to the use of excess tissue (resection surplus) for research.

### Histology

The routine diagnostic haematoxylin and eosin (H&E) stained tissue section of the sampled background liver from each of the obtained resected liver specimen was assessed for steatosis. This is expressed as a percentage of hepatocytes involvement by macro- and microvesicular steatosis based on the low (100×) to medium (200×) power microscopic evaluation of the liver parenchyma (Kleiner et al. 2005).

### Isolation of human hepatocytes

Liver tissue resection surplus was collected on ice and examined by a pathologist. Only tissue deemed healthy by the pathologist was used for hepatocyte isolation. Under aseptic conditions tissue was diced and washed in HBSS to remove excess blood. Briefly, tissue is minced on ice using two scalpels in a scissor motion. Tissue is diced until a slurry forms and tissue cannot be diced further (<3 mm). Tissue was transferred to a specimen container containing pre-warmed EGTA buffer (HBSS, 0.5 mM EGTA, 0.5% fatty acid free bovine serum albumin (BSA)) and agitated (100 rpm) in a water bath with shaking bed for 10 min, 37 °C. Tissue was then washed three times in HBSS to remove remaining blood and EGTA. Tissue was then placed in pre-warmed digestion buffer (HBSS, 0.05% collagenase IV, 0.5% fatty acid free BSA, 10 mM CaCl_2_) and agitated (100 rpm) in a water bath with shaking bed for 30 min, 37 °C. BSA was included in all digestion buffers to minimise cell damage and prevent hemolysis of red blood cells (RBCs). Digested tissue was passed through a metal (tea) strainer and supernatant collected and filtered through 100 µm cell strainer. Supernatant was kept on ice; the remaining tissue was again digested in fresh digestion buffer. Cell suspensions were pooled and centrifuged (80 g for 5 min, 4 °C) and the supernatant discarded. The hepatocyte pellet was gently re-suspended in minimal amount of liquid. As there was a large RBC component to the hepatocyte cell pellet RBC lysis buffer (RBCly) was added to hepatocyte suspension and left at room temperature for 3 min before inactivation of RBCly with PBS, cells were then centrifuged (80 g for 5 min, 4 °C). Hepatocytes were washed twice in William’s E buffer and cells counted and viability measured by trypan blue exclusion. Cells were diluted to 1 million cells per ml in William’s E buffer containing supplements (1% non-essential amino acids (NEEA), 1% GlutaMAX™, 2% human serum, 100 nM dexamethasone, 100 nM insulin and 0.375% fatty acid free BSA). Hepatocytes were plated on type 1 collagen coated plates, at a density of 250,000/cm^2^. After cells had adhered (3–4 h) media was removed and replaced with William’s E maintenance media (1% NEEA, 1% GlutaMAX™, 100 nM dexamethasone, 100 nM insulin and 0.375% fatty acid free BSA).

### Immunoblotting

Protein concentrations were determined using the BCA protein assay. Briefly, 15–20 μg whole cell lysates were subjected to SDS-PAGE using NOVEX 4–20% precast gels. Polyvinylidene fluoride (PVDF) membranes were probed with primary antibodies raised against the protein of interest. Detection of primary antibodies was performed using appropriate peroxidise-conjugated IgG and protein signals visualized using enhanced chemiluminescence and exposure to autoradiographic film. Quantification of immunoblots was done using Image J (NIH, Bethesda, MD, http://rsb.info.nih.gov/ij).

### Urea and intracellular triglyceride (TG) concentrations

Urea and TG were analyzed using Instrumentation Laboratory kits on an ILab 650 Clinical Chemistry analyzer as previously described (Green et al. 2015). Serum free and media containing FBS or lysis buffer were used as background controls.

### Morphology

Isolated hepatocytes were cultured for 16 h before washing three times in PBS. Cells were then stained for living mitochondria using TMRM. Cells were then stained for 30 min at 37 °C 5% CO2 using 50 nm TMRM in hepatocyte culture media. The staining media was removed and replaced with normal culture media. Mitochondria were then imaged using a TRITC filter on a Leica DMIRE2 microscope using a 40× objective.

### Statistics

For single comparisons non-paired *t*-tests were used and associations between variables were carried out using Spearman’s rank correlation. For cross-group analysis non-parametric one-way Anova with Dunn’s multiple comparisons were performed. Data analysis was performed using GraphPad Prism 7 software and considered statistically significant at *P* < 0.05.

## Results

We report here the isolation of primary human hepatocytes from 44 liver resections. Patients (23 males and 21 females) from which resection tissue was obtained had an average age of 61 years (range 32–78 years) and average BMI of 25.9 kg/m^2^ (range 18.3–41.9 kg/m^2^). The majority of resections were carried out due to colorectal metastases (n = 29) but also included cholangio-carcinoma or other primary liver cancer (n = 5) or had another diagnosis (n = 10). Patient gender, BMI, age and primary diagnosis had no effect on viability of isolated hepatocytes (Table 1).

**Table 1:** Overview of cell viability by tissue characteristics

*Cell viability by gender*			
Gender	Male (n = 23)	Female (n = 21)	
Viability (%)	70.5 ± 19.2	72.7 ± 10.9	
*Cell viability by liver tissue sample weight*			
Tissue weight (g)	<50 g (n = 18)	51–150 g (n = 13)	>150 g (n = 13)
Viability (%)	65.2 ± 12.3	77.9 ± 11.3**	79.0 ± 8.90**
*Cell viability by patient age*			
Age (years)	32–48 (n = 7)	49–60 (n = 10)	>60 (n = 27)
Viability (%)	73.0 ± 13.0	75.1 ± 12.0	69.9 ± 18.3
*Cell viability by patient diagnosis*			
Diagnosis	Benign/other (n = 10)	Primary/cholangio carcinoma (n = 5)	Colorectal metastisis (n = 29)
Viability (%)	78.2 ± 9.5	67.6 ± 16.9	69.05 ± 16.1
*Cell viability by BMI*			
BMI (kg/m^2^)	18–24.9 (n = 24)	25–29.9 (n = 11)	>30 (n = 8)
Viability (%)	71.6 ± 17.4	75.1 ± 9.4	66.4 ± 18.7
*Cell viability by liver steatosis grade*			
Steatosis grade (%)	No steatosis 0–5% (n = 24)	Mild steatosis 6–20% (n = 7)	Severe steatosis >20% (n = 8)
Viability (%)	73.7 ± 10.3	74.9 ± 11.1	69.4 ± 19.6

Viability data presented as mean ± SD

** *P* < 0.01 for comparison of tissue weights >50 g versus all tissue weights <50 g

**Fig. 1:**
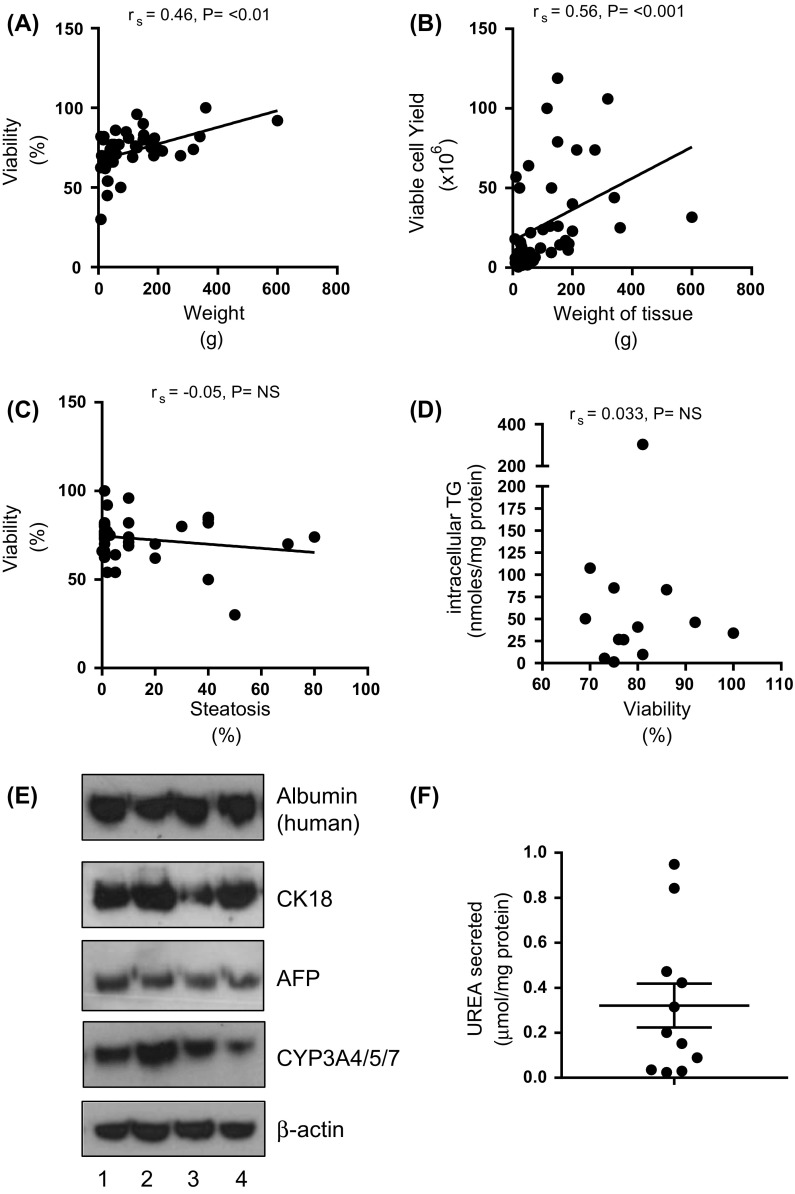
Correlations between **a** total weight of liver tissue and hepatocyte viability (%) (r_s_ = 0.46, *P* < 0.001, n = 44); **b** total weight of liver tissue and hepatocyte yield (viable cells only, ×10^6^) (r_s_ = 0.56, *P* < 0.001, n = 44); **c** histologically graded steatosis (%) and hepatocyte viability (%) (r_s_ = −0.05, *P* = NS, n = 39); and **d** intracellular triglyceride (TG) content of isolated hepatocytes after 24 h in culture (nmoles/mg protein) and percentage viability of hepatocytes following isolation (r_s_ = 0.03, *P* = NS, n = 12). **e** Western blot analysis of 4 separate hepatocyte cell isolations (1–4). β-actin was used to ascertain equal gel loading; and **f** the concentration of urea in cell medium after 24 h culture of hepatocytes [µmol/mg protein of cells in the well above background (n = 10)]. Background taken as fresh medium that had not been cultured with cells

In order to establish if the total liver tissue weight used in the digestion process influenced isolated hepatocyte viability we assessed the relationship between the total liver tissue weight of each isolation with the corresponding percentage viability of isolated hepatocytes. We found a positive correlation between the weight of tissue and percentage of viable hepatocytes (r_s_ = 0.46, *P* < 0.01, n = 44) (Fig. 1a). To establish the optimal total liver tissue weight required for isolation of viable hepatocytes we grouped the total liver tissue weight per isolation into three categories: <50 g (n = 18), 51–150 g (n = 13) and >150 g (n = 13). Hepatocytes isolated from total liver tissue weights >50 g had significantly (*P* < 0.01) higher viability than hepatocytes from <50 g of tissue (Table 1), although the mean viability of the latter was still >65%.

It is plausible that larger tissue weights would result in a greater hepatocyte cell yield. We assessed the association between the total liver tissue weight and total viable hepatocyte cell yield following isolation and found a positive correlation between tissue weight and hepatocyte yield (r_s_ = 0.56, *P* < 0.001) (Fig. 1b). Previous work has suggested isolated hepatocytes from steatotic livers are less viable than those isolated from steatosis free tissue (Alexandrova et al. 2005; Lee et al. 2014), however we found no association between grade of tissue steatosis (assessed histologically) and hepatocyte viability (r_s_ = −0.05, *P* = NS, n = 39) (Fig. 1c; Table 1). In line with this, intracellular TG concentration of the isolated hepatocytes (measured at 24 h post seeding) was not related to cell viability (r_s_ = 0.03, *P* = NS, n = 13) (Fig. 1d). However, we observed that after the plating of hepatocytes, viable cells that remained floating in the media had approximately a three-fold higher TG concentration than adherent cells (data not shown).

In order to confirm that isolated cells were hepatocytes we measured the expression of key hepatocyte proteins by western blot. Albumin (human specific) and cell surface marker cytokeratin 18 (CK18) were highly expressed after 24 h of cell culture (Fig. 1e). Hepatocytes also expressed some alpha-fetoprotein (Fig. 1e) but importantly were lacking expression of the biliary cell marker cytokeratin 19 (CK19) (data not shown). Hepatocytes retained expression of cytochrome P450 3A4/5/7 after culture for 24 h (Fig. 1e). As a readout of hepatocyte function the amount of urea in the cell medium was measured and all hepatocyte isolations measured had urea in the media above background concentrations (Fig. 1f).

**Fig. 2:**
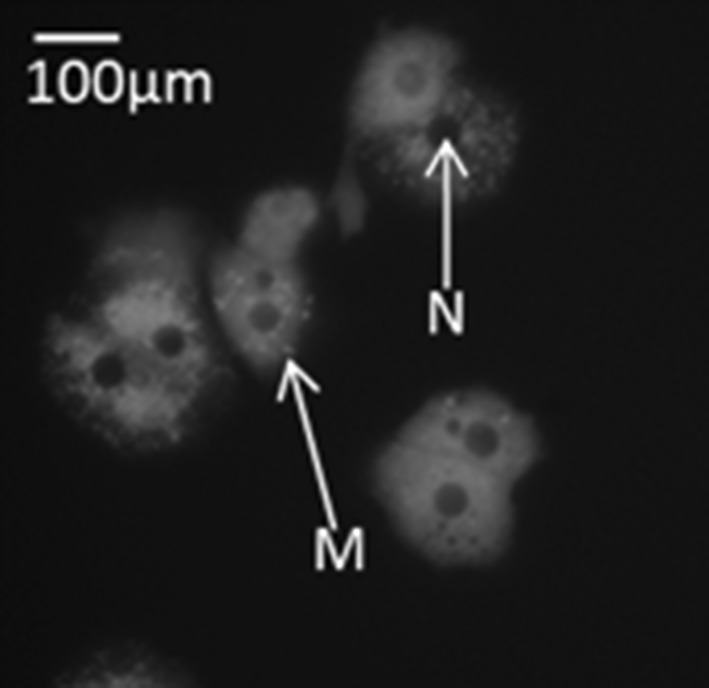
Representative ×40 magnification of primary hepatocytes stained with TMRM mitochondrial stain. Mitochondria (*M*) are indicated by *red* punctae and an example shown using the *arrow* labelled *M*. The nucleus (*N*) of the hepatocytes in indicated using the *arrow* labelled *N*. The *scale bar* represents 100 µm. (Color figure online)

The morphology of the isolated primary human hepatocytes was assessed using TMRM mitochondrial stain 16 h post tissue isolation (Fig. 2). TMRM is taken up by active mitochondria with a membrane potential and is a good indication that cells are viable. We found the primary hepatocytes contained living mitochondria and had the characteristic cuboidal shape and centrally located nucleus (Fig. 2).

## Conclusions

Primary human hepatocytes are the gold standard in vitro cell model as they retain functionality over short time courses and are thought to most closely recapitulate in vivo liver metabolism and function (Green et al. 2015). However, due to limitations in accessing appropriate liver tissue their use is often restricted. We report here methodology to isolate human hepatocytes from healthy liver resection surplus tissue which would have been unsuitable for perfusion. Our method utilises similar buffers to the two-step perfusion method (EGTA and collagenase IV) and requires RBCs to be lysed following tissue digest to circumvent contamination of RBCs in hepatocyte preparations. We demonstrate that despite using significantly smaller pieces of liver tissue, we consistently obtain viable cells with high yields (>65%). Although we did not do a direct comparison with the two-step perfusion method, taking information from work using this method (Bhogal et al. 2011; Lee et al. 2014) the time required to isolate hepatocytes from liver tissue using the method described here comparable, or slightly faster than the two-step perfusion technique.

A number of factors, including age and gender of donor, presence of steatosis, fibrosis and cirrhosis, chemotherapy and perfused liver weight have all be suggested to effect the viability and yield of isolated hepatocytes (Bhogal et al. 2011; Lee et al. 2014). Lee et al. (2014) isolated primary human hepatocytes using the two-step perfusion method, with modifications, from remnant liver pieces from 1034 patients and they found that the yield of hepatocytes was decreased by the presence of liver fat and a Ludwig score indicating septal fibrosis and the weight of resected or perfused tissue. We observed a significant positive correlation between total liver tissue weight and total yield of viable cells. Bhogal et al. (2011), using the two-step perfusion method previously reported average total viable yields of 350,000 cells. Using our methodology we found an average total yield of viable hepatocytes to be 28,000,000 cells, which is 80 times higher than that reported by Bhogal et al. (2011). In contrast, others have reported viable yields between 2.6 × 10^6^ and 18.7 × 10^6^/g tissue (Alexandre et al. 2002; Alexandrova et al. 2005; Vondran et al. 2008; Kluge et al. 2016) using perfusion whilst we report here an average viable cell yield of 0.64 ± 0.19 × 10^6^/g tissue. The discrepancy in reported yields between studies using perfusion and our methodology may be in part explained by the efficiency of perfusion, collagenase used, and the quality of liver tissue.

Factors that have been suggested to influence viability of isolated primary hepatocytes include the presence of fibrosis, steatosis and bilirubin content (Lee et al. 2014). We found a positive correlation between cell viability and liver tissue weight with > 50 g yielding significantly higher hepatocyte viabilities than weights <50 g. This observation fits with that of Bhogal et al. (2011) who reported that the optimal tissue weight for liver perfusion is 80–120 g. The reasons for this are unclear but it is potentially due to the smaller pieces of tissue having a smaller surface area, therefore more cells would be exposed to the surface resulting in a higher proportion of cells dying due to direct exposure to the environment, cold storage and digestion buffers. In contrast, larger tissues specimens would theoretically have proportionally fewer exposed cells and therefore result in higher viable cell yields following isolation. In the present work, we found that only 4% of isolations with tissue weights between 9 and 27.9 g resulted in cell viability <50%; the overall mean viability was 73 ± 13% (mean ± SEM). Our mean viability is comparable to Vondran et al. (2008) who reported 70% mean viability but is higher than that reported by others at 40% (Bhogal et al. 2011), 59% (Alexandre et al. 2002), and 64% (Alexandrova et al. 2005) all of whom used two-step perfusion. In order to increase the viability of cell isolations percol density gradients have been utilised by some (Alexandre et al. 2002; Vondran et al. 2008; Bhogal et al. 2011) however, this appears to result in a decrease (ranging from 20 to 70%) in hepatocyte cell yields (Alexandre et al. 2002; Vondran et al. 2008; Bhogal et al. 2011).

We found the amount of tissue steatosis or TG within the hepatocytes had no effect on the viability of the cells, which is consistent with the findings by some (Alexandre et al. 2002; Bhogal et al. 2011) but not all (Alexandrova et al. 2005; Vondran et al. 2008; Lee et al. 2014). Tissue steatosis has been reported to affect adherence of viable cells (Bhogal et al. 2011). We found that non-adherent cells, although viable, had a higher TG content than adherent cells. Based on our observations, it is plausible to suggest that hepatocytes from steatotic liver tissue may need to be seeded at higher densities or left for longer before medium changes for adherence to occur.

In conclusion we report here a non-perfusion method for the isolation of viable human hepatocytes, from healthy liver resection surplus tissue. Human primary hepatocytes are considered the gold standard in vitro cellular model to study human liver function and metabolism. The methodology described here which offers the opportunity to increase the use of resection surplus tissue for hepatocyte isolation, may ultimately lead to an increased number of in vitro cellular studies being undertaken using human primary hepatocytes.

## References

[CR1] Alexandre E, Cahn M, Abadie-Viollon C, Meyer N, Heyd B, Mantion G, Cinqualbre J, David P, Jaeck D, Richert L (2002). Influence of pre-, intra- and post-operative parameters of donor liver on the outcome of isolated human hepatocytes. Cell Tissue Bank.

[CR2] Alexandrova K, Griesel C, Barthold M, Heuft HG, Ott M, Winkler M, Schrem H, Manns MP, Bredehorn T, Net M, Vidal MM, Kafert-Kasting S, Arseniev L (2005). Large-scale isolation of human hepatocytes for therapeutic application. Cell Transplant.

[CR3] Berry MN, Friend DS (1969). High-yield preparation of isolated rat liver parenchymal cells: a biochemical and fine structural study. J Cell Biol.

[CR4] Berry MN, Simpson FO (1962). Fine structure of cells isolated from adult mouse liver. J Cell Biol.

[CR5] Bhogal RH, Hodson J, Bartlett DC, Weston CJ, Curbishley SM, Haughton E, Williams KT, Reynolds GM, Newsome PN, Adams DH, Afford SC (2011). Isolation of primary human hepatocytes from normal and diseased liver tissue: a one hundred liver experience. PLoS ONE.

[CR6] Green CJ, Johnson D, Amin HD, Sivathondan P, Silva MA, Wang LM, Stevanato L, McNeil CA, Miljan EA, Sinden JD, Morten KJ, Hodson L (2015). Characterization of lipid metabolism in a novel immortalized human hepatocyte cell line. Am J Physiol Endocrinol Metab.

[CR7] Guguen-Guillouzo C, Campion JP, Brissot P, Glaise D, Launois B, Bourel M, Guillouzo A (1982). High yield preparation of isolated human adult hepatocytes by enzymatic perfusion of the liver. Cell Biol Int Rep.

[CR8] Howard RB, Pesch LA (1968). Respiratory activity of intact, isolated parenchymal cells from rat liver. J Biol Chem.

[CR9] Howard RB, Christensen AK, Gibbs FA, Pesch LA (1967). The enzymatic preparation of isolated intact parenchymal cells from rat liver. J Cell Biol.

[CR10] Jacob ST, Bhargava PM (1962). A new method for the preparation of liver cell suspensions. Exp Cell Res.

[CR11] Kegel V, Deharde D, Pfeiffer E, Zeilinger K, Seehofer D, Damm G (2016). Protocol for isolation of primary human hepatocytes and corresponding major populations of non-parenchymal liver cells. J Vis Exp.

[CR12] Kleiner DE, Brunt EM, Van Natta M, Behling C, Contos MJ, Cummings OW, Ferrell LD, Liu YC, Torbenson MS, Unalp-Arida A, Yeh M, McCullough AJ, Sanyal AJ, Nonalcoholic Steatohepatitis Clinical Research N (2005). Design and validation of a histological scoring system for nonalcoholic fatty liver disease. Hepatology.

[CR13] Kluge M, Reutzel-Selke A, Napierala H, Hillebrandt KH, Major RD, Struecker B, Leder A, Siefert J, Tang P, Lippert S, Sallmon H, Seehofer D, Pratschke J, Sauer IM, Raschzok N (2016). Human hepatocyte isolation: does portal vein embolization affect the outcome?. Tissue Eng Part C Methods.

[CR14] Lecluyse EL, Alexandre E (2010). Isolation and culture of primary hepatocytes from resected human liver tissue. Methods Mol Biol.

[CR15] Lee SM, Schelcher C, Demmel M, Hauner M, Thasler WE (2013). Isolation of human hepatocytes by a two-step collagenase perfusion procedure. J Vis Exp.

[CR16] Lee SM, Schelcher C, Laubender RP, Frose N, Thasler RM, Schiergens TS, Mansmann U, Thasler WE (2014). An algorithm that predicts the viability and the yield of human hepatocytes isolated from remnant liver pieces obtained from liver resections. PLoS ONE.

[CR17] Longmuir IS, Rees WA (1956). Preparation of cell suspensions from rat livers. Nature.

[CR18] Pfeiffer E, Kegel V, Zeilinger K, Hengstler JG, Nussler AK, Seehofer D, Damm G (2015). Featured article: isolation, characterization, and cultivation of human hepatocytes and non-parenchymal liver cells. Exp Biol Med (Maywood).

[CR19] Seglen PO (1972). Preparation of rat liver cells. I. Effect of Ca 2+ on enzymatic dispersion of isolated, perfused liver. Exp Cell Res.

[CR20] Vondran FW, Katenz E, Schwartlander R, Morgul MH, Raschzok N, Gong X, Cheng X, Kehr D, Sauer IM (2008). Isolation of primary human hepatocytes after partial hepatectomy: criteria for identification of the most promising liver specimen. Artif Organs.

[CR21] Werner M, Driftmann S, Kleinehr K, Kaiser GM, Mathe Z, Treckmann JW, Paul A, Skibbe K, Timm J, Canbay A, Gerken G, Schlaak JF, Broering R (2015). All-in-one: advanced preparation of human parenchymal and non-parenchymal liver cells. PLoS ONE.

